# Quantifying the heritability of belief formation

**DOI:** 10.1038/s41598-022-15492-0

**Published:** 2022-07-12

**Authors:** Valentina Vellani, Neil Garrett, Anne Gaule, Kaustubh R. Patil, Tali Sharot

**Affiliations:** 1 Affective Brain Lab, Department of Experimental Psychology, University College London, London, WC1H 0AP UK; 2 The Max Planck UCL Centre for Computational Psychiatry and Ageing Research, University College London, London, WC1B 5EH UK; 3 School of Psychology, University of East Anglia, Norwich Research Park, Norwich, NR4 7TJ UK; 4 Developmental Risk and Resilience Unit, Department of Experimental Psychology, University College London, London, WC1H 0AP UK; 5 Institute of Neuroscience and Medicine (INM-7), Forschungszentrum Jülich, Jülich, Germany; 6 Institute of Systems Neuroscience, Medical Faculty, Heinrich Heine University Düsseldorf, Düsseldorf, Germany; 7 Department of Brain and Cognitive Sciences, Massachusetts Institute of Technology, Cambridge, MA USA

**Keywords:** Behavioural genetics, Cognitive neuroscience, Human behaviour

## Abstract

Individual differences in behaviour, traits and mental-health are partially heritable. Traditionally, studies have focused on quantifying the heritability of high-order characteristics, such as happiness or education attainment. Here, we quantify the degree of heritability of lower-level mental processes that likely contribute to complex traits and behaviour. In particular, we quantify the degree of heritability of cognitive and affective factors that contribute to the generation of beliefs about risk, which drive behavior in domains ranging from finance to health. Monozygotic and dizygotic twin pairs completed a belief formation task. We first show that beliefs about risk are associated with vividness of imagination, affective evaluation and learning abilities. We then demonstrate that the genetic contribution to individual differences in these processes range between 13.5 and 39%, with affect evaluation showing a particular robust heritability component. These results provide clues to which mental factors may be driving the heritability component of beliefs formation, which in turn contribute to the heritability of complex traits.

## Introduction

A significant proportion of human variations in personality, behaviour and well-being is heritable^1–10^. Quantifying the degree of heritability of different traits has been of avid interest for decades (for review see ^11^). A meta-analysis of nearly 3000 twin studies found that estimates of heritability are vastly different across domains^11^. For example, the degree of heritability of weight maintenance is much larger than of conduct disorder^11^. Such knowledge is important as it can inform the development of interventions to alter the phenotype.

For complex traits related to psychological functions heritability estimates are around 50% on average^11^. For example, more than half of the total variance in intelligence^1^ and nearly half of the total variance in personality^2^ are explained by genetic differences. Similar estimates are found for heritability of psychiatric conditions^6,7^.

Yet, despite the vast literature quantifying the heritability of high-level personality traits (such as extraversion^2^) and behaviours (such as participation in the stock market^8^), little is known of the degree of heritability of the basic cognitive and affective mechanisms that contribute to them. While such quantification is challenging as it requires administering long multi-trial tasks to large samples of twins, it is crucial for identifying the factors that most contribute to the heritability of complex traits.

Here, we set out to quantify the degree of heritability of basic mental processes that contribute to the formation of beliefs about future outcomes. We are constantly flooded with information that forms our beliefs about the world. Beliefs, in turn, drive our actions^12^, decisions^13^ and mood^14^. We zoomed in on the formation of beliefs about personal risk (e.g., the likelihood of being infected by a virus; the likelihood of losing money) because of the large impact of such beliefs on individuals’ behaviour and mental health^15^.

To examine whether people are endowed with genetic differences that could explain individual variation in the processes contributing to belief formation, we administrated a belief formation task to Monozygotic (MZ) and Dizygotic (DZ) same-sex twin pairs. MZ twins are identical twins who share 100% of their genes. DZ twins are non-identical twins who share 50% of their genes. Heritability can be assessed by comparing how similar MZ and DZ twins are in relation to a phenotype^16^.

Within the task we measured the heritability of cognitive and affective mental processes that have been known to influence belief formation, including: affective assessment^17–20^, learning^21^, memory^22–25^, and vividness of imagination^26,27^. Learning was measured by quantifying an individual’s tendency to alter existing beliefs in response to new information. A recall test was administered to measure participants’ ability to accurately recollect such information. Participants were also asked to imagine the event in question and then asked to assess how vivid the image was in their minds and how negatively arousing it was. When events are imagined with increased vividness, people tend to predict their likelihood of happening as greater^28^. Affective assessment has also been associated with beliefs, with negative events predicted to be less likely to occur^22^. We quantified the extent to which these four mental processes (learning, memory, vividness of imagination, affective evaluation) are heritable in the context of risk assessment, while controlling for phenotypes of no interest, including participants’ familiarity and past experience with the events.

## Result

### Beliefs are associated with vividness of imagination, affective assessment, learning and memory.

**Figure 1:**
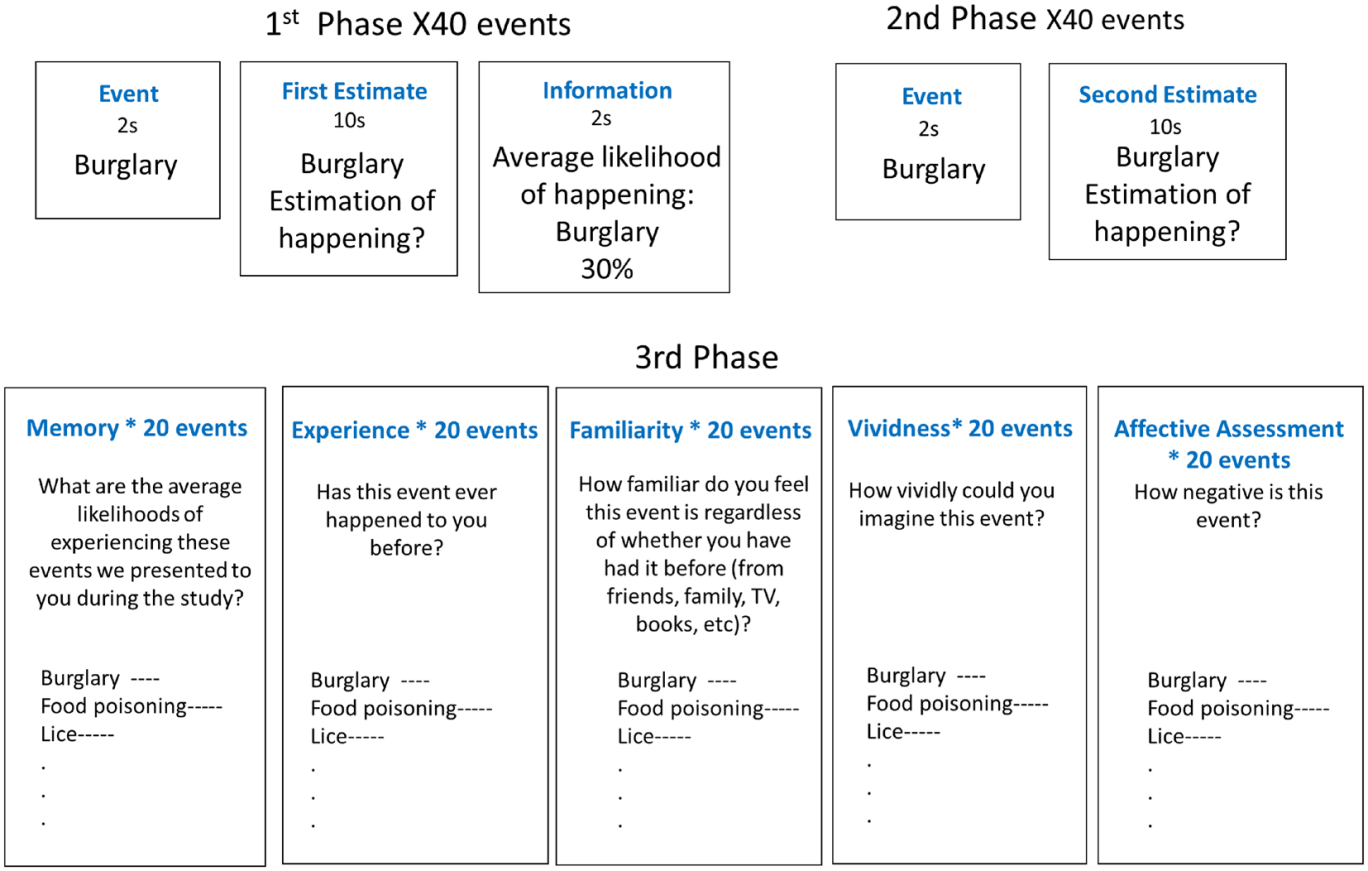
Belief Formation Task. On each trial, participants were presented with a short description of an adverse life event (2 s) and asked to estimate how likely this event was to occur to them in the future. They had up to 10 s to respond. They were then presented with the probability of that event occurring to someone from the same age, location and socio-economic background as them for 2 s. This was repeated for 40 different events. The second phase was the same as the first except that the average probability of the event to occur was not presented. In the third phase, subjects were asked to recollect the probability previously presented of the events and rate the events on past experience, familiarity, vividness of imagination and affective assessment. The last phase was repeated for 20 events randomly selected out of the 40 events.

In this study we measured participants’ (n = 528) beliefs of their likelihood of experiencing future adverse events, and how those were altered in response to new information and related to a range of cognitive and affective process. The task (see Fig. 1) was adapted from previous studies^17,18,22^. Participants were presented with 40 adverse life events (e.g., robbery, card fraud) and asked to estimate how likely the event was to happen to them in the future (this is referred to as the first estimate or prior). They were then presented with the base rate of the event in a demographically similar population (this is referred to as information). In a second phase, participants were asked again to provide estimates of their likelihood of encountering the same events (this is referred to as the second estimate or posterior). In the third phase they observed all events again and asked to rate them on vividness, affective assessment, as well as familiarity and past experience. Additionally, their memory for the information presented in the first phase of the study was tested.

**Figure 2:**
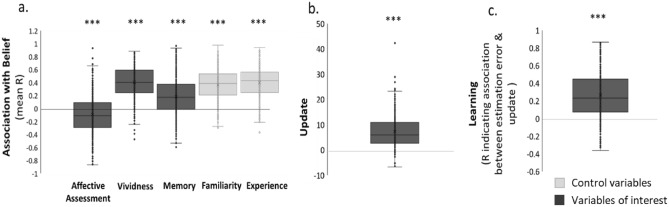
Beliefs are associated with vividness of imagination, affective assessment, learning and memory. (**a**) Plotted are the mean correlation coefficients obtained by correlating for each participant across trials their prior belief (first estimate) with each rating. Results show that participants believed they were more likely to encounter an aversive event when they imagined the event more vividly, less negatively, and also when they were more familiar with the event, and had experienced it in the past. Participants also tended to remember the likelihood presented to them as greater when their prior regarding that likelihood was greater. (**b**) Average update scores (calculated as the difference between the first and second estimate coded positively when update is towards the information and negatively otherwise) were significantly greater than zero, suggesting that beliefs are modulated in response to new information. (**c**) Learning scores (the correlation between update and estimation errors) are significantly greater from 0, indicating that subjects were learning. Horizontal lines indicate median values, boxes indicate 25–75% interquartile range, the crosses indicate mean values and whiskers indicate 1.5 × interquartile range; individual scores are shown separately as circles.***p < 0.001. In black are variables of interest for heritability quantification.

We first sought to examine if participants’ prior beliefs about their likelihood of experiencing aversive events were indeed associated with vividness of imagination and affective assessment. As predicted, participants believed they were more likely to encounter an aversive event when they imagined the event more vividly (M = 0.41, SD = 0.23, t(516) = 40.364, *p* < 0.001) and less negatively (M = − 0.09, SD = 0.30, t(514) = 7.107, *p* < 0.001). In addition, they predicted the event to be more likely to occur when they were more familiar with the event (M = 0.37, SD = 0.23, t(522) = 36.376, *p* < 0.001) and had experienced it in the past (M = 0.40, SD = 0.23, t(526) = 39.505, *p* < 0.001). Interestingly, participant’s memory of the information they were shown was also correlated with their prior belief (M = 0.18, SD = 0.30, t(527) = 13.921, *p* < 0.001), suggesting that priors influence subsequent memory for information. The above statistics were obtained for every rating scale by conducting a Pearson correlation for each participant between their prior belief (that is their first estimate) and each rating across trials. Pearson Coefficients were then compared to zero across participants with a t-test (Fig. 2a). Similar results are obtained when entering all subjective ratings simultaneously as independent variables to compete for variance, and prior beliefs as the dependent variable in a mixed model with fixed and random main effects and intercepts. Again, we find that people believe an event is more likely to occur if they have experienced it before (β = 4.367 ± 0.573 (SE), t(178.857) = 7.628, *p* < 0.001), are familiar with it form the experience of others (β = 2.250 ± 0.450 (SE), t(325.785) = 4.995, *p* < 0.001), imagine the event with great vividness (β = 2.661 ± 0.431 (SE), t(434.601) = 6.173, *p* < 0.001) and assess the event as less negative (trend level: β = − 0.655 ± 0.383 (SE), t(561.942) =  − 1.712, *p* = 0.087). This analysis assesses the contribution of each variable while controlling for all other variables and suggests that these variables have independent contributions to participants’ prior estimates.

To test for learning, we first calculated the magnitude of belief change on each trial (that is, the difference between the first and second estimate), which we refer to as ‘update’. This was coded positively if the update was in the direction of the information received and negatively otherwise. Update (M = 7.23, SD = 6.13, t(527) = 27.104, *p* < 0.001) was significantly greater than zero, suggesting that beliefs are modulated in response to new information (Fig. 2b). Second, we calculated an estimation error term on each trial, which is the absolute difference between participants’ first estimate and the probability presented (information). We correlated these with update for each individual across trials to generate a “learning score”. Results show that across participants’ the learning score (M = 0.27, SD = 0.26, t(527) = 23.908 *p* < 0.001) was significantly greater than zero, suggesting that subjects were learning (Fig. 2c).

### Quantifying the degree to which cognitive and affective factors associated with belief formation are heritable.

Thus far, we have shown that participants’ beliefs are associated with their learning ability, memory, vividness of imagination and affective assessment. Next, we turned to the main aim of the study, which was to examine to what extent these processes are heritable.

The 528 participants who completed the study were same-sex twin pairs (80 dizygotic twin pairs and 184 monozygotic twin pairs). There was no significant difference between dizygotic (DZ) and monozygotic (MZ) twins in terms of gender (DZ: females = 86.2%, MZ: females = 88.9%; X^2^ (1) = 0.773, p = 0.379). DZ twins were slightly older than MZ twins (DZ: mean age = 55.31, SD = 12.31; MZ: mean age = 50.26 SD = 14.86; t(522) = 3.767, *p* < 0.001). We therefore control for age in all twin analysis. Age or gender were unknown for three twin pairs, thus their data was not included in the twin analysis.

We first examined at the difference in correlation between MZ twins and DZ twins for each variable. Specifically, we computed the correlation between monozygotic twin A and twin B on a phenotypee (*rMZ*) and the correlation between dizygotic twin A and twin B on a phenotype (*rDZ*). Since assignment of twins in each pair to the abscissa (i.e., twin A) or ordinate (i.e., twin B) is arbitrary and this assignment influences the correlation value, we performed a permutation analysis. Specifically, we randomly reassigned each twin in the pair as twin A or twin B 10,000 times, each time obtaining a correlation coefficient for the monozygotic samples and for the dizygotic sample^29^.

Each correlation (i.e., each iteration of *rMZ* and *rDZ*) was in fact a partial correlation that controlled for the twin’s age, first estimate (prior), all subjective ratings (past experience, familiarity, vividness, and affective assessment) and memory score (calculated as % of trials in which information was accurately remembered). Because first estimate (prior) is provided before information is revealed we did not control for memory when analyzing first estimate. For belief updating we also controlled for subjects’ estimation errors. Adding these controls allowed us to examine the heritability of the phenotype of interest beyond the heritability of related constructs. Not controlling for these factors does not alter the results (see Supplementary Table 1).

As detailed below, for all variables the 95% confidence intervals of the partial Pearson correlation coefficient for MZ twins and the partial Pearson correlation coefficient for DZ twins did not overlap, with the former being larger than the latter. This suggests that for all variables the scores of MZ twins are more tightly associated than for DZ twins.

**Figure 3:**
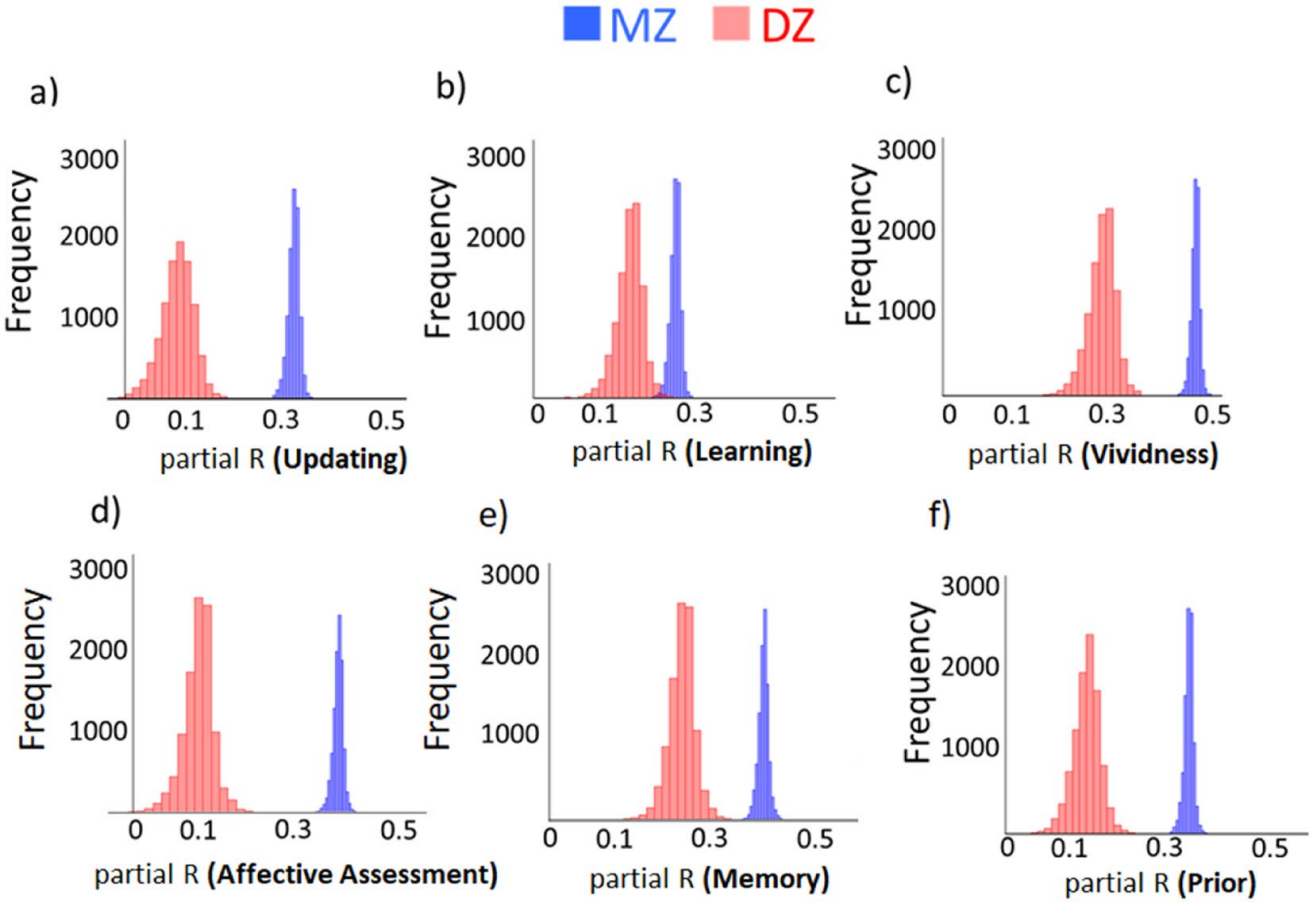
Distribution of cognitive and affective factors associated with beliefs. Plotted are the distributions of the partial correlation (controlling for all covariates—this means that in an analysis of one factor above we are controlling for all the other above factors) between monozygotic (blue) twin A and twin B and dizygotic (red) twin A and twin B for each variable of interest. Since assignment of twins in each pair to the abscissa or ordinate is arbitrary and this assignment influences the correlation value, we randomly reassigned each twin in the pair as twin A or twin B 10,000 times, each time obtaining a correlation coefficient for the monozygotic samples (blue) and for the dizygotic one (red). A stronger correlation for monozygotic twins’ than dizygotic twins indicate heritability of the phenotype. This is indeed observed for belief updating, learning, memory, vividness of imagination, affective assessment, and priors (i.e. first estimate). Note that the width of the distribution for MZ twins is smaller than for DZ because the N is larger.

**Figure 4:**
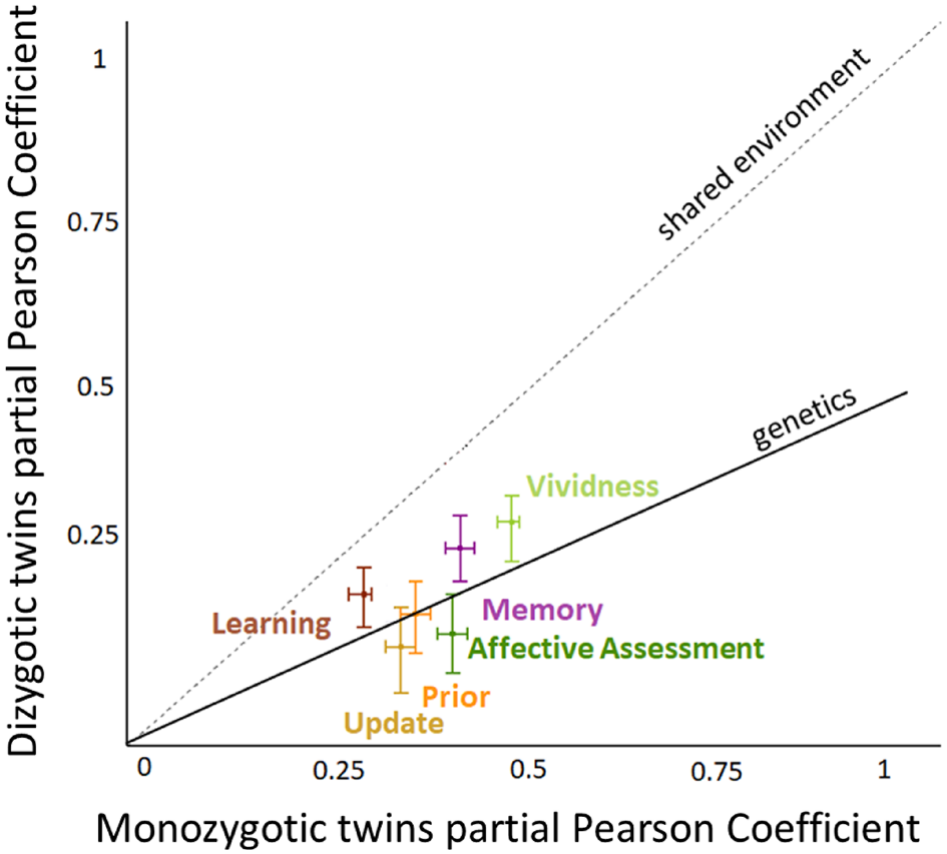
Plotted on the Y axis is the mean partial Pearson coefficient between DZ twins. Plotted on the X axis is the mean partial Pearson coefficient between MZ twins. Each cross represents a phenotype, clearly marked on the graph. The size of the cross’s line represents the the 2.5% and 97.5% estimates of the distribution. If the similarity on a phenotype within a twin pair is explained by shared environment alone than the mean coefficient should be the same for MZ twins and DZ twins. In such cases the cross should fall on the dotted line. If the similarity on a phenotype within a twin pair is also explained by genetics than the mean coefficient should be larger for MZ twins than DZ twins and fall near the dotted line. The closer the cross is to the bold line the greater the impact of heritability on the phenotype. The partial coefficient are calculated while controlling for all the other variables, as well as past experience and familiarity. Thus, each represents a heritability estimates for a specific factor independent of the rest.

We had two measures of people’s tendency to incorporate new information into their beliefs—update and learning score. For belief updating (Figs. 3a and 4) mean R coefficient was 0.32 (2.5%/97.5% = 0.30/0.34) for MZ pairs, and 0.10 (2.5%/97.5% = 0.03/0.16) for DZ pairs. For learning score (Figs. 3b and 4), the mean R coefficient for MZ pairs was 0.27 (2.5%/97.5% = 0.25/0.28) and 0.18 (2.5%/97.5% = 0.13/0.22) for DZ pairs.

For affective assessment (how negative events were perceived to be), (Figs. 3d and 4) the mean R coefficient was 0.39 (2.5%/97.5% = 0.37/0.41) for MZ twin pairs and 0.12 (2.5%/97.5% = 0.06/0.18) for DZ twin pairs. For first estimate (prior) (Figs. 3f and 4) mean R coefficient for MZ pairs was 0.34 (2.5%/97.5% = 0.32/0.36) and 0.15 for DZ pairs (2.5%/97.5% = 0.09/0.20).

For self-reported vividness in imagining events (Figs. 3c and 4) the mean R coefficient was 0.47 (2.5%/97.5% = 0.45/0.48) for MZ twin pairs and 0.29 (2.5%/97.5% = 0.23/0.33) for DZ twin pairs. For memory scores (Figs. 3e and 4) mean R coefficient was 0.40 (2.5%/97.5% = 0.38/0.42) for MZ twin pairs and 0.25 (2.5%/97.5% = 0.20/0.30) for DZ twin pairs.

We next fit the data to the ACE model^3,4,31–39^ to assess the genetic contribution of the cognitive and affective factors contributing to belief formation. The ACE model is an epidemiological model stating that genetic factors (A), common environmental factors (C) and specific environmental factors (E) explain individual differences in a given phenotype. The model enables us to quantify the contribution of each factor by comparing the covariance of scores in the phenotype of interest across monozygotic and dizygotic twins. We also computed partial models that dropped either the ‘A’ (additive genetic) component, the ‘C’ (shared environment) component, or both.

Moreover, for each variable we also computed the ADE model. In the ADE model, the common environment factor has been replaced by the dominance genetic factor (D). Note, that according to the literature DE is not estimated since it is not possible to estimate dominant genetic effects without additive ones^16,30^.We also compared goodness of fit, as assessed by the AIC score for the ADE, ACE model and partial models.

When running the models we control for age, gender, interaction between age and gender, first estimate, all subjective ratings (past experience, familiarity, vividness, and affective assessment) and memory. As first estimate is given before information is received we do not control for memory scores when analysing first estimate. When analysing update scores we also control for estimation errors. Not controlling for these factors does not change the results (see Supplementary Table 1).

For both update and learning the best fitting model was the AE model which explains variance taking into account heritability and specific environment. For learning, the additive heritability estimate was 15.3% (CI: 1.3/28.6%) while specific environment accounted for 84.7% (CI: 71.4/98.7%) of the variation in the phenotype (Table 1). For update, the heritability estimate was 13.1% (CI: 1.6e-14/28%). The lower CI was close to zero and thus this effect is only marginal. Specific environment accounted for 86.9% (CI: 72/100%) of the variation (Table 1).

**Table 1:** ACE and ADE Estimates. In BOLD is the estimates of the winning model for each phenotype.

Variable	Model	A (CI)	D(CI)	C (CI)	E (CI)	AIC	Minus2LL	ep	df
Memory	AE	38.8% (26.6/49.7)			61.2% (50.3/73.4)	2692.631	2686.631	3	519
	ACE	24.3% (5.6e-12/49.2)		13.6%(4.7e-15/43.0)	62.1% (50.8/75.0)	2694.247	2686.247	4	518
	ADE	38.8% (1.7e-15/49.7)	1.7e-11%(1.9e-22/47.1)		61.2% (50.3/75.0)	2694.631	2686.631	4	518
	CE			34.3%(23.2/44.6)	65.7% (55.4/76.8)	2693.365	2687.365	3	519
	E				100%	2724.068	2720.068	2	520
Affective Assessment	AE	35.2% (22.1/47.1)			64.8% (52.9/78.0)	1466.472	1460.472	3	519
	ACE	35.2% (8.9/47.1)		5.12e-11 (2.8e-14/21.6)	64.8% (52.9/78.0)	1468.472	1460.472	4	518
	ADE	2.6e-13% (4.1e-15/44.7)	37.2%(1.2e-11/48.9)		62.8% (51.1/76.0)	1467.015	1459.015	4	518
	CE			26.6%(15.0/37.5)	73.4% (62.5/85)	1472.275	1466.275	3	519
	E				100%	1418.588	1414.588	2	520
Vividness	AE	28.1% (15.0/40.1)			71.9% (59.9/85.0)	1403.637	1397.637	3	519
	ACE	28.1% (1.6e-16/40.1)		2.7e-13%(2.7e-13/26.2)	71.9% (59.9/85.0)	1405.637	1397.637	4	518
	ADE	1.1% (4.9e-14/38.4)	29.5%(3.9e-22/41.5)		70.5% (58.5/83.7)	1404.609	1396.609	4	518
	CE			22.5%(10.6/33.6)	77.5% (66.3/89.4)	1407.089	1401.089	3	519
	E				100%	1418.588	1414.588	2	520
Priors	AE	27.1% (13.0/40.1)			72.9% (59.9/87.0)	3554.813	3548.813	3	519
	ACE	27.1% (3.1e-15/40.1)		5.7e-15 (8.4e-19/23.5)	72.9% (59.9/87.0)	3556.813	3548.813	4	518
	ADE	3.4e-11% (3.8e-15/38.5)	29.3%(1.4e-12/42.3)		70.7% (57.7/85.3)	3555.869	3547.869	4	518
	CE			19.6%(7.7/30.9)	80.4% (69.1/92.3)	3558.239	3552.239	3	519
	E				100%	3566.434	3562.434	2	520
Learning	AE	15.3% (1.3/28.6)			84.7% (71.3/98.7)	-61.60747	-67.60747	3	519
	ACE	15.3% (1.5e-14/28.6)		2.8e-12%(3.4e-13/22.2)	84.7% (71.4/98.7)	-59.60747	-67.60747	4	518
	ADE	2.7e-14% (4.2e-19/28.2)	16.2%(1.9e-16/30)		83.7% (70.0/98.24)	-59.83635	-67.83635	4	518
	CE			11.9%(2.2e-20/23.6)	88.1% (76.4/100.0)	-60.70621	-66.70621	3	519
	E				100%	-58.99647	-62.99647	2	520
Update	AE	13.1% (1.6e-14/28.0)			86.9% (72.0/100)	3241.202	3235.202	3	519
	ACE	13.1% (3.1e-15/28.0)		3.1e-13 (2.5e-19/14.7)	86.9% (72.0/100)	3243.202	3235.202	4	518
	ADE	9.8e-12% (2.2e-18/25.1)	17.2%(1.6e-14/32.5)		82.8% (67.5/99.3)	3241.814	3233.814	4	518
	CE			6.1%(4.2e-17/18.0)	93.9% (82.0/100)	3243.046	3237.046	3	519
	E				100%	3242.01	3238.01	2	520

(A) donates the estimated contribution of heritability, (E) of specific environment, and (C) of common environment to the phenotype. In the ADE model, the common environment factor has been replaced by the dominance genetic factor (D). Akaike Information criterion (AIC) is a measure of goodness of fit which penalize for number of parameters. A lower number suggests a better fit. Also shown is the negative Log likelihood (-2ll), the number of parameters including the intercept (ep) and the degrees of freedom (df). Estimates are calculated controlling for other phenotypes, age, gender and their interaction. Not controlling for these factors does not change the results (see Supplementary Table 1). Variables are ordered from highest to lowest variability explained by genetics.

A significant genetic contribution was found both for vividness of imagination and subjective assessment of negativity. For both variables, the best fitting model was the AE model which explains variance taking into account additive heritability and specific environment. For vividness, the heritability estimate was 28.1% (CI: 15/40.1%), while specific environment accounted for 71.9% (CI: 59.9/85%) of the variation in the phenotype (Table 1). For assessment of negativity, the heritability estimate was 35.2% (CI: 22.1/47.1%), while specific environment accounted for 64.8% (CI: 52.9/77.9%) of the variation in the phenotype (Table 1).

For memory scores the results suggest that the best fitting model was the AE model. Additive heritability estimate was 38.8 (CI: 26.6/49.7%), while specific environment accounted for 61.2% (CI: 50.3/73.4%) of the variation in the phenotype (Table 1). For priors, the best fitting model was also the AE model which explains variance taking into account heritability and specific environment. The heritability estimate was 27.1% (CI: 13/40.1%) while specific environment accounted for 72.9% (CI: 59.9/87%) of the variation (Table 1).

The stimuli presented to the subjects included some conditions with known genetic predispositions (such as medical conditions) and some that are less likely to have genetic predispositions (such as having ants in the house). We thus looked separately at medical conditions with known genetic predisposition and other events with no known genetic predispositions. We found that the heritability estimates of participants’ prior beliefs did not differ for these types of events. The best fitting model was the AE model for for both those events with and without known genetic predisposition. In particular, for those events with known genetic predispositions, additive heritability estimate was 32.7 (CI: 19.4/44.8%), while specific environment accounted for 67.2% (CI: 55.2/80.6%) of the variation in the phenotype. For events without known genetic predispositions, additive heritability estimate was 19.1 (CI: 4.4/33%), while specific environment accounted for 80.8% (CI: 67/95.6%) of the variation. This suggests that participants’ *perception* of risk is heritable regardless of the heritability of the event itself.

The results thus far suggest that cognitive and affective factors that underlie belief formation (including learning, vividness of imagination and affective assessment) are heritable. This is true even when controlling for the twins’ familiarity and past experience with the events as well as their first estimates, which is heritable, and all other variables.

## Discussion

To what extent is variation in human behaviour due to ‘nature’ and ‘nurture’? This century old question^40–43^ is still occupying psychologists, philosophers, biologists, and economists today^1–12,34,34,36–39^. Quantifying the extent of genetic influence is important, partially because it has implication for how and when interventions are likely to be effective.

Human action is guided by beliefs about what the future holds. One set of beliefs that is key to people’s well-being^14,15^ and decisions^12,13^ in domains ranging from finance to health are beliefs about future harm. For example, beliefs about one’s likelihood of being infected by COVID-19 is related to mitigating behaviour^44^, and beliefs about the likelihood of a market collapse is related to investment and consumption^13^.

Here we quantify the degree of genetic contribution to variations in basic cognitive and affective factors associated with beliefs about future harm. Importantly, we first show that people believe they are more likely to encounter an aversive event when they can imagine it more vividly and estimate it as less negative. We also show that people learn well from new information, updating their beliefs appropriately. We then find that individual variation across all of these processes can be partially explained by genetics to varying degrees. The degree to which genetics plays a role in these processes ranges from approximately 13% to 39%.

Estimates were obtained for each factor while controlling for variation in the other factors. We also control for people’s familiarity with the events and their direct past experience with the events. Therefore, heritability estimates are unlikely to be due to heritability of related constructs.

Our results provide novel insight into the degree to which basic mental processes are heritable. In so doing we provide clues to the likely degree of heritability of high order characteristics. For example, the AE model indicated that affective evaluation was one of the most heritable factors we tested (35%). It is possible that basic functions such as affective evaluation can explain the large heritability found for high-level variations, such as in mental health. In contrast, the heritability estimate of learning was relatively low (AE: 15.3%), while those of vividness and memory were both in the 30 s as was for belief about risk itself. Interestingly, the degree of heritability of risk estimates were the same regardless of whether the risk itself was highly heritable (such as the risk of experiencing cancer) or not (such as being burgled). This suggests that the *perception* of risk is heritable. However, it is important to recognize that generalizability of genetic studies is limited. For example, it is possible that cultural factors might act as modulating factors^46,47^, therefore, future studies could investigate whether the results reported in this paper remain stable across cultures. Moreover, power to estimate the genetic component, while within the normal range for twin studies, is not high (especially for learning and update) and studies with even larger samples would be helpful.

Most studies^1,3–12,32,37–39^ have focused on quantifying the heritability of high order traits and behaviours. By quantifying lower-level cognitive and affective mechanism, we may be able to draw general conclusions about the likely degree of heritability of a large range of behaviours and traits and identify the factors that contribute most to such heritability. Such knowledge can guide attempts to modify a phenotype, as an intervention may be more successful if it relies on a process that is more sensitive to context than genetics.

## Materials and Methods

### Participants

528 twins completed the study. 484 were recruited via Twins UK and 44 via a list maintained by a lab at UCL. Most subjects provided demographics information (99.2% provided age, 98.8% gender). All participants filled in the informed consent. Subjects recruited via the UCL list were paid for their participation. The study was approved by the departmental ethics committee at UCL. All experiments were performed in accordance with relevant guidelines and regulations of the UCL ethics committee.

Sample size is based on previous studies investigating heritability of individual differences in human behavior^36–38,48–50^. The sample was composed of 264 twin couples (80 dizygotic twin couples and 184 monozygotic twin couples). Smaller DZ than MZ samples is common in the literature^36–38,48–50^ due to identical twins being more motivated to participate in twin research. Estimates regarding DZ twins may thus be less precise than MZ twins. Nonetheless, our DZ sample size is equivalent to that used previously to investigate the genetic component of psychological constructs^37,38,48–51^. Because our design is within subject repeated measures, the variables of interest are obtained by averaging across many trials. This provides more power than twin designs in which only trial one measurement is taken (such as a single question). Indeed, other studies using a repeated measure design typically use a DZ sample size ranging from 64 to 214^36,48–51^. The common equation to assess power in twin designs^52^ was not developed to assess power in repeated measures design. However, using this equation suggest power estimates for estimating the genetic component (A) for past repeated measures twin studies range between 0.24 and 0.88^36,48–51^ and between 0.12 and 0.85^37,38^ for non-repeated designs testing psychological variables. The equivalent power in the current study lays firmly within this range (0.51–0.28) for memory, affect, vividness and priors, but on the lower end (0.15–0.14) for learning and update.

#### Paradigm

Participants performed a formation task online using Qualtrics. The task was adapted from past studies^17,18^. All subjects completed a practice session of three trials before beginning the main experiment. On each trial one of 40 adverse life events were presented for 2 s. Participants were asked to estimate how likely the event was to happen to them in the future. Participants had up to 10 s to respond. If they failed to respond within 10 s an error message appeared asking them to insert a valid response. It was not possible to continue until a valid response was inserted. Then, participants were presented with the probability of an event occurring in a demographically similar population for 2 s. In a second session, immediately after the first, participants were asked again to provide estimates of their likelihood of encountering the same events so that we could assess how they updated their estimate in response to the information presented. Probabilities of the events occurring were not provided in this second session (Fig. 1).

#### Stimuli

Forty short descriptions of negative life events (for example: domestic burglary, card fraud) were presented in a random order. Very rare or very common events were not included; all event probabilities lay between 10 and 70%. To ensure that the range of possible overestimation was equal to the range of possible underestimation, participants were told that the range of probabilities lay between 3 and 77% and were only permitted to enter estimates within this range. For each adverse event, the average probability of that event occurring in a demographically similar population was determined from online resources^19^ (Office for National Statistics, Eurostat, PubMed).

#### Memory Scores

To measure recollection participants were asked at the end of the experiment to provide the actual probability previously presented. Due to time constraints imposed by Twins UK, participants provided the actual probability for 20 of the 40 events, randomly selected. Percentage of correct recollection was calculated for each subject.

#### Ratings

After completing the task, participants also rated the events on prior experience, familiarity, vividness and affective assessment. Due to time constraints imposed by Twins Uk participants rated 20 events of the 40 events (randomly selected) on familiarity [‘‘How familiar do you feel this event is regardless of whether you have had it before (from friends, family, TV, books, etc.)?’’ on a scale from 1 (not at all familiar) to 6 (very familiar)], prior experience [‘‘Has this event ever happened to you before?’’ on a scale from 1 (never) to 6 (very often)], vividness [“How vividly could you imagine this event?” (1, not at all vivid, to 6, very vividly)] and negativity [‘‘How negative is this event?’’ on a scale from 1 (not negative at all) to 6 (very negative)].

### Statistical analysis

#### Task variables

Update was calculated on each trial as the difference between the subject’s first estimate and second estimate. This quantity was given a positive sign when the change was towards the probability presented and negative sign when the change was in the opposite direction of the probability presented. We then computed the average update for each participant. To calculate learning scores, an estimation error for each trial was computed by taking the absolute value of the difference between subjects’ first estimate and the information provided. Then, for each subject we correlated the update and the estimation error across all trials. One-sample t-tests were performed to assess whether participants’ scores in belief updating and learning were different from 0, which would indicate update and learning was in the direction of the information presented rather than reflecting random change. For each subject a memory score was computed as the percentage of correct responses across trials. For all other scales we simply computed the average response for each subject across trials.

Then we assess whether subjects’ first estimates were related to their ratings (past experience, familiarity, affective assessment, vividness, and memory) and their memory of what information they were shown. For each participant we correlated across trials the first estimate and each ratings. We then performed a one-sample t-tests to assess whether the correlation coefficients obtained were different from 0. In addition, to assess whether the ratings were independently related to the first estimate, we performed a Mixed Model with first estimate as the dependent measure and the ratings as the independent measure. We included all main fixed and random effects and a fixed and random intercept.

#### Assessing genetic contribution

We first computed, for each twin, the phenotypes of interest as described above. We then examined the relationship between the phenotypes between twins. Specifically, a partial correlation was computed between twin pairs (i.e. twin A’s phenotype scores were correlated with twin B’s phenotype scores), separately for DZ twin pairs and MZ twin pairs. The partial correlations controlled for age, first estimate, memory, vividness rating, affective assessment, past experience and familiarity with the event. For update we also controlled for estimation error. Because first estimate (prior) is provided before information is revealed we did not control for memory when analyzing first estimate.

The control variables were calculated as follows:

$${\text{Control factor}}_{{\text{j}}} {\text{averaged over all trials twin A {-} Control factor}}_{{\text{j}}} {\text{averaged over all trials twin B}}$$

Since labelling each twin in a pair as twin A or twin B is arbitrary but impacts the correlation value, we performed a permutation analysis. Before computing each partial correlation, we randomly assigned each twin in a pair as A or B. We repeated this process 10,000 times, obtaining 10,000 correlation coefficients for monozygotic twins and 10,000 correlation coefficients for dizygotic twins^29^ for our phenotypes. For all variables we tested whether the 95% confidence intervals of the partial Pearson correlation coefficient for MZ twins and the partial Pearson correlation coefficient for DZ overlapped.

#### ACE and ADE Analysis

We used the ACE model^3,4,31–39^ to assess the genetic contribution to the phenotypes of interest.

The ACE model is an epidemiological model stating that genetic factors (A), common environmental factors (C) and specific environmental factors (E) explain individual differences in a given phenotype. The model enables us to quantify the contribution of each factor by comparing the covariance of scores in the phenotype of interest across MZ twins and DZ twins.

The ACE model is formally expressed as:

$$y_{ij} = \mu + A_{ij} + C_{j} + E_{ij}$$

where y is the phenotype of interest (update in response to positive information or negative information in our case), j denotes the dyad, i the twin within the dyad, µ the mean of the phenotype across all observations, A_ij_ is the genetic component, C_j_ is the shared environment component and E_ij_ is the specific environment component. This implies the assumption that the variance of a given phenotype is explained as the sum of the variance of the genetic, shared and specific environmental factors.

$$Var\left( y \right) = \sigma_{A}^{2} + \sigma_{C}^{2} + \sigma_{E}^{2}$$

The ACE model also assumes that:

- Common environment is entirely shared between twins (COV(C_1j_, C_2j_) = 1).
- Specific environment is unique for each twin (COV(E_1j_, E_2j_) = 0).
- Monozygotic twins share 100% of their genes (COV_MZ_(A_1j_, A_2j_) = σ_A_^2^), while dizygotic twins share 50% of their genes (COV_DZ_(A_1j_, A_2j_) = *½* σ_A_^2^).

From these assumptions two equations are obtained:

$$COV_{MZ} \left( {y_{1j} , \, y_{2j} } \right) = \sigma_{A}^{2} + \sigma_{C}^{2}$$

$$COV_{DZ} \left( {y_{1j} , \, y_{2j} } \right) = \raise.5ex\hbox{$\scriptstyle 1$}\kern-.1em/ \kern-.15em\lower.25ex\hbox{$\scriptstyle 2$} \sigma_{A}^{2} + \sigma_{C}^{2}$$

Based on these equations, it is possible to estimate the ACE model using a random effects regressions model with 2 × 2 covariance-variance matrix as the following (R is the genetic relatedness of the twin pair which is equal 1 for MZ twins and *½* for DZ twins.):

$$\Omega j = \left[ {\begin{array}{*{20}l} {\sigma_{A}^{2} + \sigma_{C}^{2} + \sigma_{E}^{2} \;\;\;R_{j} \sigma_{A}^{2} + \sigma_{C}^{2} } \hfill \\ {R_{j} \sigma_{A}^{2} + \sigma_{C}^{2} \;\;\;\sigma_{A}^{2} + \sigma_{C}^{2} + \sigma_{E}^{2} } \hfill \\ \end{array} } \right]$$

We used the variance of the random effects to generate the estimates of genetic, common environment and specific environment factors. The structural equation-modelling program OpenMx^45^, implemented in R, was used to estimate the three factors. Moreover, 3 additional submodels were estimated:

- The AE model, according to which genetics and the specific environment determine the phenotype (C = 0).
- The CE model, according to which the common and the specific environment determine the phenotype (A = 0).
- The E model, according to which only the specific environment determines the phenotype (A = 0 and C = 0).

We also run the ADE mode where the common environment factor has been replaced by the dominance genetic factor (D). We also tested the partial models that dropped either the ‘D’ (dominance genetic) component, or bot the ‘D’ and ‘A’ components. Since it is not possible to estimate dominant genetic effects without additive ones, DE models are not estimated^16^.

Thus, for each variable ACE, ADE, CE and E models were computed. We then compared the fit of the models to the data of each variable using the Akaike Information criterion (AIC), where smaller AIC scores indicate better fit. The AIC has been calculated as following:$AIC = - 2LL + 2*ep.$

In all models we control for age, gender, the interaction between gender and age first estimate, memory, vividness rating, affective assessment, past experience and familiarity with the event. For update we also controlled for estimation error and we did not control for memory when analyzing first estimate.

## Supplementary Information

Supplementary Information.

## Data Availability

Data and code available on Github: https://github.com/affective-brain-lab/TwinStudy_UpdateBiasTask_2020.
