# Supplementary material for: Quantifying the heritability of belief formation
Source: Sci Rep. 2022 Jul 12;12:11833. doi: 10.1038/s41598-022-15492-0 (PMC9276818; doi:10.1038/s41598-022-15492-0)
Supplement: Supplementary file 1 — Supplementary Information. [file 41598_2022_15492_MOESM1_ESM.docx]

**Supplementary Materials**

Below we report all estimates calculated with no controls.


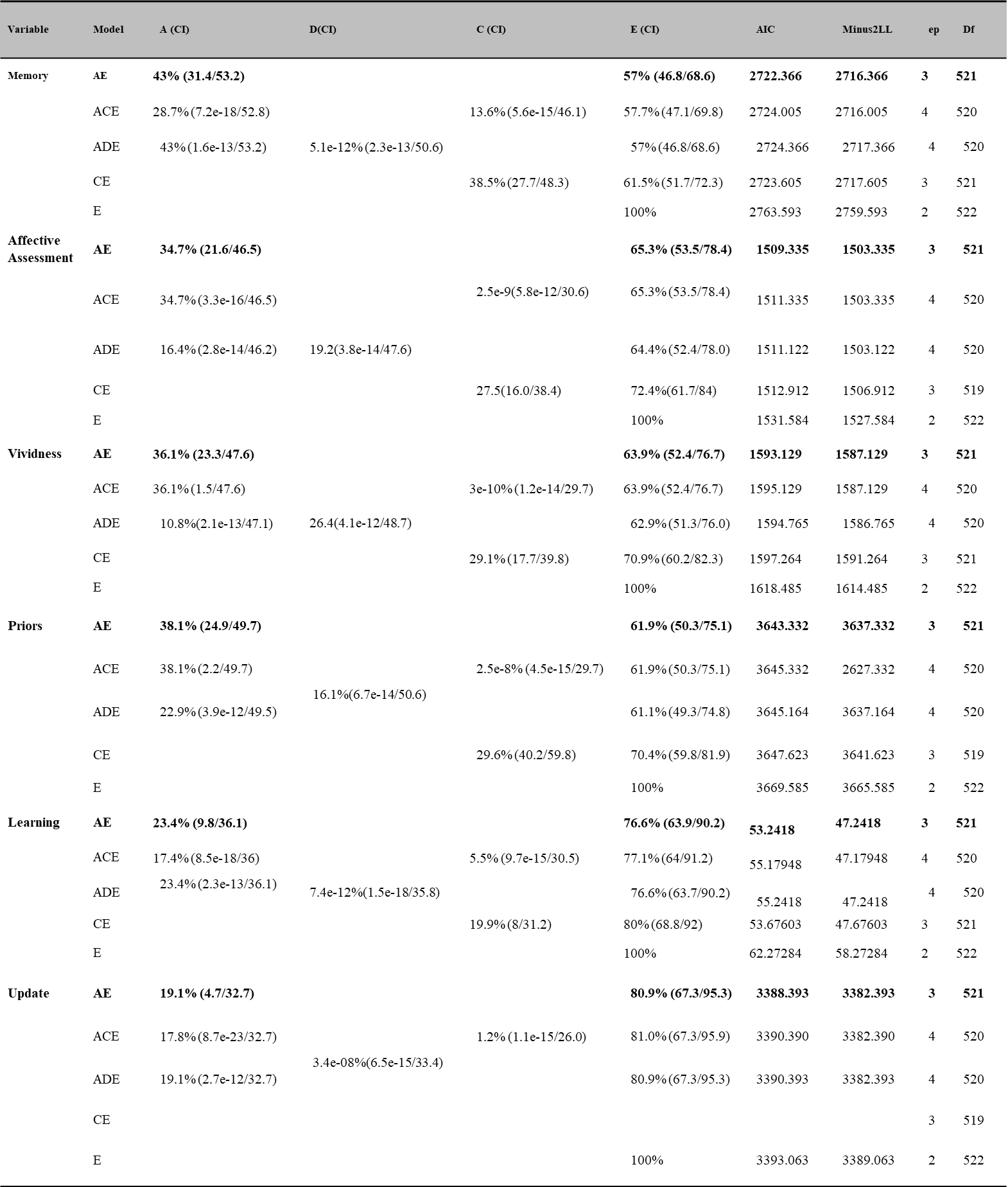


**Table 1. ACE and ADE Estimates.** In BOLD is the estimates of the winning model for each phenotype. (A) donates the estimated contribution of heritability, (E) of specific environment, and (C) of common environment to the phenotype. In the ADE model, the common environment factor has been replaced by the dominance genetic factor (D). Akaike Information criterion (AIC) is a measure of goodness of fit which penalize for number of parameters. A lower number suggests a better fit. Also shown is the negative Log likelihood (-2ll), the number of parameters including the intercept (ep) and the degrees of freedom (df).

**Bivariate AE Analysis**

**Methods**

We examined whether genetic effects of the high-level variable (belief, that is first estimate) overlapped with the genetic effects of the other variables (affective assessment, vividness, memory, learning and updating). To do so, we performed a bivariate twin analysis using the structural equation-modelling program OpenMx^45^, implemented in R. Bivariate models examine the extent to which genetic and environmental factors explain the correlation between two phenotypes, that is, they allow to assess how much of the phenotypic correlation between the traits is accounted for by genetic and environmental factors.

Because the univariate models suggested that the best fitting model was AE for all variables, we performed a bivariate AE model to estimated genetic and environmental contributions to the covariance between belief and each of the other variable.

**Results**

Results of the bivariate twin analysis revealed that for first estimate and vividness, the bivariate heritability was 51.4% (CI: 16.7/83.1) and 48.6% (CI: 16.9/85.3%) of covariance was explained by unique environmental effects. For first estimate and belief updating, the bivariate heritability was 33.1% (CI: 15.4/49.2), and 66.9% (CI: 15.4/84.6%) of covariance was explained by unique environmental effects. For first estimate and learning, the bivariate heritability was 45.4% (CI: 27.6/61.7) and 54.6% (CI: 38.3/72.4%) of covariance was explained by unique environmental effects. For first estimate and affective assessment and first estimate and memory the bivariate heritability was higher than 100%, suggesting the model did not fit the data.
